# Who support open access publishing? Gender, discipline, seniority and other factors associated with academics’ OA practice

**DOI:** 10.1007/s11192-017-2316-z

**Published:** 2017-03-06

**Authors:** Yimei Zhu

**Affiliations:** School of Media, Communication and Sociology, University of Leicester, 132 New Walk, Leicester, LE1 7JA UK

**Keywords:** Open access, Open science, Gold OA, Green OA, Academic publishing, Scholarly communication

## Abstract

This paper presents the findings from a survey study of UK academics and their publishing behaviour. The aim of this study is to investigate academics’ attitudes towards and practice of open access (OA) publishing. The results are based on a survey study of academics at 12 Russell Group universities, and reflect responses from over 1800 researchers. This study found that whilst most academics support the principle of making knowledge freely available to everyone, the use of OA publishing among UK academics was still limited despite relevant established OA policies. The results suggest that there were differences in the extent of OA practice between different universities, academic disciplines, age and seniorities. Academics’ use in OA publishing was also related to their awareness of OA policy and OA repositories, their attitudes towards the importance of OA publishing and their belief in OA citation advantage. The implications of these findings are relevant to the development of strategies for the implementation of OA policies.

## Introduction

The development of World Wide Web and other new technology has changed the manner in which people access and disseminate scientific knowledge. Over the last decade, academic publication in the UK has shifted from hard copy print to online journals, which has liberated researchers from the physical library and shifted the potential barrier to the access to those online journals (Nicholas et al. 2009). Open access (OA) publishing emerged in the internet age which includes two types of electronic publishing on a website (Swan and Brown 2004). Authors can provide open access to their research articles by publishing in open access journals or by self-archiving articles in online repositories (Björk et al. 2010; Correia and Teixeira 2005). The earliest open access journals were founded in the early 1990s (Björk 2004). By 2004, there were over 100 universities worldwide who had Open Access Eprint Archives (Harnad et al. 2004). Since then, both institutional and disciplinary OA repositories have continued to grow. OA policies have also been discussed and adopted to certain extent by various academics, funders and institutions in Western countries. However, OA policies had not been mandatory in the UK. Only recently in July 2014, academics were required to publish articles through open access channels if they were subject to the next *Research Excellence Framework* (REF) (HEFCE 2015). Little is known to what extent UK academics have been practising OA publishing and who those early adopters were. This paper aims to explore the extent of support and use of OA publishing and the characteristics and factors associated with the use of OA publishing. It will give recommendation to universities, stake holders and academics for a better implementation of OA policies and science advancement.

## Literature review

Open access publishing has been a much discussed mechanism for an open science approach over the last decade. One major rationale of open access publishing is that the public should have free access to publicly funded research. Because a paywall leads to a situation where the public cannot use restricted reliable information in the form of peer reviewed academic research and instead may trust potentially unreliable information from the Internet (Gray 2009). Open access to scientific publications can help the public access reliable information from research outputs. Another rationale of OA publishing is related to the publishing crisis and the criticism of publishers making profit from academic publishing. Academic libraries face a crisis that has continued for many years (McGuigan and Russel 2008). Academic authors and scholars usually provide the content as well as peer reviewing and editorial services to scholarly journals and they are commonly unpaid by the journals. The universities purchase the journals and pay salaries to academic authors and editors whilst the publishers make profit on providing access to the journals. After the shift of paper format to electronic format, publishers of academic journals started to offer site licenses for online access to academic institutions (Björk 2004). Since there is very little competition in academic publishing, the subscription price continues to rise. In protest against the high subscription costs of mainstream publishers, a growing number of researchers, institutions and funding bodies have started open access journals and online repositories which are freely accessible online (Björk 2004).

The two main open access channels are commonly referred to as Gold OA and Green OA. Gold OA refers to online journal articles which are either totally or to some extent made freely accessible to the public by the publishers (Laakso et al. 2011). Most Gold OA publishers require Article Processing Charge (APC) which cost authors approximately 3000 USD for their articles to be published with unlimited online access (Björk et al. 2010; Willinsky 2010). Green OA refers to the self-archiving of an author’s research article or an earlier version of the manuscript (Crow 2008; Laakso 2014). Green OA articles are supplied by the authors on a web site that is freely available without publisher mediation (Pinfield 2003). Research articles can be deposited in institutional repositories, subject-based repositories or authors’ own websites (Björk 2004).

OA publishing not only benefits the general public by offering free access of scientific content but also provides potential benefits for individual authors. The most discussed potential advantage of OA publishing is related to OA articles’ citation impact. Since the first study by Lawrence (2001) suggested a citation advantage of OA articles, many studies on OA articles’ citation impact have been conducted (Craig et al. 2007). Earlier studies of citation impact have mostly focused on the effect of Green OA and generally indicated that self-archiving of scientific publications can lead to increases in the number of article citations (Metcalfe 2005, 2006; Schwarz and Kennicutt 2004). Other studies of Green OA suggested that early view effects and self-selection of posting high quality work are responsible for the citation effect rather than OA status (Davis and Fromerth 2007; Henneken et al. 2006; Kurtz et al. 2005; Moed 2007). As for Gold OA, a number of studies found OA advantage while comparing OA articles with non-OA articles from the same journal (Davis 2009; Eysenbach 2006; Sotudeh et al. 2015; Wang et al. 2015). Others found little evidence for citation impact of Gold OA articles after taking account of a full set of control variables (Calver and Bradley 2010; Gaule and Maystre 2011). Gaule and Maystre (2011) suggested that OA citation impact might be explained by selection bias as authors of higher quality papers were more likely to choose open access in hybrid journals. Selection bias was also suggested by McCabe and Snyder (2014), who analysed a panel of science journal articles moving from paid to open access after controlling for article quality. They identified a small citation impact of OA status for top-ranked journals, but citation deduction for bottom-ranked journals. No citation advantage was found by Davis and colleagues, who designed a randomised controlled trial which randomly assigned OA status to articles in various online subscription-based journal articles and eliminated the influence of the early view effects and selection bias (Davis 2011; Davis and Walters 2011). However, OA availability was found to have led to more downloads (Davis 2010; Davis et al. 2008). Regardless of the cause for OA articles’ citation advantage, most studies suggest that OA status may contribute to a higher visibility and more readerships.

In the UK, a number of research councils have had open access policies since 2005. From April 2013, Research Council UK’s (RCUK) open access policy came into effect which required RCUK-funded research to be published either through open access journals or self-archiving (RCUK 2013). RCUK arranged to pay block grants to universities and other institutions to support the cost of APC for publishing in Gold OA journals. Many universities also started institutional repositories to support Green OA model (e.g. PURE for University of Bristol^1^ and ORA for Oxford University^2^). In July 2014, the Higher Education Funding Council for England (HEFCE) also introduced an open access policy which applies to research outputs accepted for publication after 1 April 2016 in relation to the research assessments after the 2014 REF.

However, OA publishing had not been a norm for academics. Back in 2006 many academics seemed to be unaware of the concept of open access or chose to remain ignorant of its implications in spite of having heard of the term (Swan 2006). A number of studies of institutional repositories across the United States, West Europe and Australia suggest that academics had little awareness of opportunities for self-archiving in digital repositories, and that a large percentage of documents in those repositories were deposited by a librarian or administrative staff (Kim 2011; Xia 2007; Xia and Sun 2007). By 2010, over 1200 online repositories were publically accessible world-wide, potentially allowing free access to at least half of current academic literature; however, authors have not participated in self-archiving in large numbers (Willinsky 2010). Björk et al. (2010) found that only 12% of all scholarly papers published in 2008 were freely accessible through some form of Green OA. Many UK based academics might not be aware of the existence of OA repositories or RCUK’s OA policy.

For Gold OA, academics were concerned that new OA journals did not have sufficient impact in their field (Swan and Brown 2004). Although major academic publishers provide OA author-pay options for those prestigious subscription based journals, the high cost of APCs is a barrier for non-funded academics. The author-pays model can also lead to quality control issues and poor or fake peer review process with OA journals (Bohannon 2013). An international survey of senior researchers found that authors’ major concerns regarding which journals to publish in are favourably associated with the ‘reputation of the journal’, its ‘readership’ and its ‘impact factor’ (Rowlands and Nicholas 2006). In contrast, the level of open access to a journal and the ability to deposit preprint or post-print in a repository were considered to be of relatively low importance. Hence many academics may not regard OA publishing as important. Moreover, in academia, the dominant idea has been established that science is too difficult and complex for the lay public audience to understand (Bucchi 2004). Davies (2008) also found that majority of scientists and engineers in her study viewed the constructions of science communication as one-way and negative. Hence, some scientists may hold negative views towards making articles freely accessible to the general public as scholarly articles could be misunderstood by non-academic audience.

Various studies found disciplinary differences in OA publishing. Open access journals have been well-developed in Medical and Life Sciences compared to other discipline areas (Björk et al. 2010). In Humanities, there were lower levels of availability for OA journal (Darley et al. 2014). While scientific journal articles are the dominant publication form for academics in Medical and Natural Sciences, monographs were found to have great value for career advancement and cited far more frequently than journal articles in the Arts and Humanities (Thompson 2002; Williams et al. 2009). Compared to the availability of OA journals, open access monograph is far less common and is in great need of a sustainable business model in order to survive the monograph publishing crisis (Look and Pinter 2010). With regard to Green OA model, open access repositories are more established in Natural Sciences. In Physical and Mathematical Sciences, a subject-based repository called arXiv,^3^ is frequently used for depositing research articles (Björk 2004). There are a number of established subject-based repositories in Social Sciences, such as the Social Science Research Network (SSRN) and Social Science Open Access Repository (SSOAR). Open access repositories in Humanities are less established or administrated at a small scale by individual libraries or departments.

Gender, age and seniority are likely to be correlated for academics suggested by previous studies. In academia, gender differences were evident in job status, job mobility and academic achievements when women were among the disadvantaged group (Børing et al. 2010; Fox 2001; Hopkins et al. 2013), manifesting in women being more likely to have junior jobs. Junior academics are also likely to be younger. Therefore, in academia, women are likely to be younger and at junior positions. Previous studies found age inversely associated to internet and other new media use (Dutton et al. 2005; Helsper and Eynon 2010; Nicholas and Rowlands 2011). Hence, this paper explores differences between groups of academic discipline, gender, age and seniority for supporting OA publishing. This is the first study to explore a number of UK research intensive universities for OA practice. Having reviewed relevant literature as discussed earlier, three research questions emerge:

**RQ1**: To what extent do academics support and use open access publishing?
**RQ2**: Were there differences between groups by university, academic discipline, gender, age and seniority for using OA publishing?
**RQ3**: What other factors (awareness of OA policy and OA repositories, attitude towards OA publishing, and belief in OA citation impact) are associated with academics’ use of OA publishing?

## Methods

This paper draws on data from a survey study of academics based in the UK with regards to their scholarly communication practice. The sampling frame of the survey consisted of the population of all academics based in the Russell Group universities. The population for the sample was chosen because the Russell Group universities have a strong research focus. As such the research would be capturing the attitudes and behaviour of some of the academics at leading research focused universities. Furthermore, Russell Group universities are large universities with a broad range of disciplines for both Science and Humanities (RussellGroup 2016). Hence the sample was designed to capture a representative picture of diverse disciplines in UK higher education institutions.

Clustering was used to lower the cost of distribution of the survey (Weisberg and Bowen 1977). Each university became a primary sampling unit (PSU) and half of PSUs were chosen in the sample (Moser and Kalton 2004). Ten out of twenty original Russell group universities and two out of four new group members that joined in August 2012 were randomly selected. The email addresses from these twelve universities’ official websites were captured using a script written in the Perl programming language. Perl programming is commonly used for automatic data mining on the internet (Pham and Bogdan 2010). The harvesting generated a long list of email addresses from each of the twelve universities. As the survey was targeted for academics, irrelevant email addresses for each university were deleted manually. Because of the large number of email addresses in the sample, it was not realistic to check whether each email address belonged to an academic by cross-referencing to the person’s information on his/her university website. Therefore, all the remaining email addresses were used for the sample and respondents were notified in the invitation email that the population targeted for this study was academic researchers. The final numbers of email addresses in the sample was 42,008 and an email with a link to a web-based questionnaire was sent out in June and July 2013. In total, 1829 valid responses were received with a response rate at 4.4%.

Limitation of the survey methods used in this study must be noted. As only half of the Russell Group universities were selected for the sample, the survey is limited in its representativeness for other UK higher education institutions. Moreover, exclusion bias resulted from exclusion of particular groups from the sample, such as those having no email addresses listed on their university websites and those who did not check their emails during the period of survey distribution. It is also possible that the techniques used could have failed to harvest certain email addresses from sampled universities’ websites.

Nonetheless, this survey is the largest of its kind to examine UK academics’ scholarly communication practices and the response rate is substantial for statistical analysis. Web-based surveys were found to have much lower response rates compared to post surveys for the same kind of study (Hardigan et al. 2012). The response rate of this study is similar to other web-based surveys for studying scholarly communication (e.g. Procter et al. 2010; Swan and Brown 2004), which indicates that 4.4% of response rate is reasonable for this type of web survey. The sample used in this study is reflective of Russell Group universities because of the selection methods used. Even though it is not a random sample, the survey has strong explanatory power and thus it is robust enough to conduct analysis. Comparing to the data sourced from the (HESA 2013), this sample was broadly representative of the broader UK academic population in terms of the weight of responses by our primary background characteristics of gender, academic discipline and age. As such, whilst there are considerable limitations to the data, the large number of responses provides a rich source of data to examine the research questions.

One section of the survey questionnaire asked respondents’ attitudes, awareness and experiences in open access publishing. Respondents were asked whether they had published articles in OA journal and if applicable, how the author fee was paid for. Furthermore, they were also asked whether they had self-archived research articles and if they were aware of open access repositories. Respondents were asked to rate the importance of making research articles freely accessible online and whether they believed that OA articles would receive a greater number of citations. Additionally, respondents were questioned whether they were aware of RCUK’s OA policy which came into effect three months before the survey was distributed. The survey responses were downloaded to Excel and analysed in SPSS.

Survey results consist of 46% female and 54% male. The disciplines in the original questionnaire were listed in the same order as the official 2014 REF categories and were grouped into four discipline areas—Medical and Life sciences (35%), Natural Sciences and engineering (23%), Social sciences (27%) and Arts and Humanities (15%). Respondents include 35% aged under 35, 26% aged 35–44, 21% aged 45–54 and 18% aged 55 and over. Job grade was categorised as researchers in training (20%) including PhD candidates, Master students and research assistants, lecturers/research fellows/postdocs (39%), senior lecturers/senior researchers (16%) and professors/readers (24%).

Descriptive statistics were applied using cross tabulations to measure the associations between academics’ use of OA publishing with key characteristics and factors (gender, age, seniority, academic discipline, awareness of RCUK OA policy and OA repositories, attitude towards the importance of OA publishing and belief in citation advantage of OA articles). A cross tabulation is a useful technique for measuring the association between two or more categorical variables, as long as each variable does not have too many categories (Lewin 2005). In addition, inferential statistics were applied to test the significance level of these associations using Chi square tests (Barnes and Lewin 2005). The Chi square test can examine whether there is a real association between two categorical variables. If the significance value *p* is smaller than 0.05, the null hypothesis that the two variables are in fact unrelated to each other can be rejected (Field 2009).

## Findings

### Overall attitudes and experience in supporting and using OA publishing

The vast majority of respondents (93%) acknowledged the importance of open access publishing, as shown in Table 1. Of the 1722 respondents, 57% rated it ‘very important’ and 36% rated it ‘fairly important’ for research articles freely to be made accessible online to everyone, while only 1 and 6% rated it ‘not at all important’ and ‘not very important’ respectively.

**Table 1:** Attitudes towards the importance of OA publishing

	*N*	%
*How important do you think it is, in general, to make research articles freely accessible online to everyone?*		
Very important	987	57
Fairly important	619	36
Not very important	104	6
Not at all important	12	1
Total	1722	100

When asked whether they had published articles in a journal that was open access, 108 respondents (6%) indicated that publishing articles was not applicable to them as they had not or did not produce research articles, and these types of responses were filtered out of the frequency, as shown in Table 2. Of the remaining respondents who had produced research articles, 41% answered ‘yes’ to having published an OA journal, 31% stated ‘no, but I plan to in the future’, 10% stated ‘no, I have no plan to publish in an OA journal’, and another 18% answered ‘no, not sure about this’.

**Table 2:** Experience of Gold and Green OA publishing

	*N*	%
*Have you published an article in a journal that is open*-*access? (Gold OA)*		
Yes	649	41
No, but I plan to in the future	501	31
No, I have no plan to publish in OA journal	163	10
No, not sure about this	288	18
Total	1601	100
*Have you deposited your research articles in an open*-*access online repository? (Green OA)*		
Yes	696	43
No, but I plan to in the future	415	26
No, I have no plan to do so	126	8
No, not sure about this	377	23
Total	1614	100
*If you have published research articles, have you had Gold or/and Green OA experience?*		
No	626	39
Gold only	276	17
Green only	326	21
Both	357	23
Total	1585	100

Of those who had produced research articles, 43% indicated their experience of depositing their papers in OA repositories. In order to evaluate the overall experience of using Gold and Green OA publishing, the two questions regarding whether respondents had published with Gold and Green OA were combined. After excluding ‘not applicable’ answers for both variables, there were 1585 respondents who had published research articles. Approximately 23% of respondents had published through both Gold and Green OA channels, 17% had published through Gold OA channel only and 21% published through Green OA channel only, and another 39% stated they had no experience in either OA channel. Thus, within the subsample of academics that had already published articles, in total 61% had experience of at least one type of open access publishing (with 38% had one of the two OA publishing experience).

After excluding respondents who had no publishing experience, the percentage of respondents who rated it ‘very important’ or ‘fairly important’ to make research articles freely accessible online was still 93%. As only 61% of those who had published articles used Gold or/and Green OA routes, there was a 32% difference between the attitudes and the reported experiences of OA publishing.

While acknowledging its importance, some respondents left comments which are related to the potential problems such as copyrights, concerns of the quality of OA journals and not being able to pay APCs. These concerns may explain the differences between attitudes regarding the importance and the reported experiences of OA publishing. For example, a senior lecturer stated her concerns about copyrights, the perceived lower quality of OA journals and not having funds to pay author fees:

In principle I am all for open access to other researchers, however in practice it is open to abuse (i.e. copyrights not always adhered to by all readers) and I also do not have the funds to pay the fees that are charged. I have also noted significantly lower quality in online access journals, where authors have to pay to get their articles published. (Female, Senior Lecturer, Biological Sciences)

Similarly, a senior lecturer in Applied Health stated his distrust of OA journals’ quality:

I do not believe that open access journals are of as good quality in terms of peer reviewing and therefore do not rate them highly. (Male, Senior lecturer, Applied Health Professions, Dentistry, Nursing and Pharmacy)

In regard to OA journals’ author fees, of the 625 respondents who had published articles in Gold OA, only 4% paid out of their own pockets for the author fees for their last OA publication, as shown in Table 3. Most respondents (66%) stated that their institutions, research funds or their collaborators paid for the APC charges. As shown in Table 4, all respondents were asked whether they preferred to publish research articles in open access journals rather than subscription-based journals if they had similar reputation or ranking of citation impact. Of the 1626 academics whose work involved publishing research papers, 39% stated that they had no preference and it was dependent on which journals had higher reputations in their field. Additionally, 31% stated that they would prefer to publish in open access journals if they personally did not need to pay the author fees. Only 9% preferred OA journals even if they personally had to pay APCs.

**Table 3:** Author fee in last Gold OA publication

	*N*	%
*If yes (having published in OA journal), did you or your institution pay for the author fee in your last OA publication?*		
Yes, my institution/research fund paid	364	58
Yes, I paid out of my own pocket	24	4
No, our collaborators paid	47	8
No, the publishers waved the fee	31	5
No, no fee was required	159	25
Total	625	100

**Table 4:** Preference of Gold OA compared to subscription based journals

	*N*	%
*In general, do you prefer to publish research articles in open access journals rather than subscription based journals if they have similar reputation or ranking of citation impact?*		
Yes, I prefer OA journals even if I personally have to pay author fee	146	9
Yes, I prefer OA journals only if I personally don’t have to pay author fee	498	31
No, I prefer conventional subscription-based journals	164	10
I don’t have a preference, it all depends on which journals have higher reputation in my field	642	39
Don’t know enough information about this matter	176	11
Total	1626	100

### Awareness of OA repositories and policy and attitudes towards OA citation impact

Table 5 suggests, of all the respondents, 42% were clear about RCUK’s open access policy which came into effect three months before the survey was conducted. Another 28% were not aware of the OA Policy and 30% indicated ‘heard of, but not sure about the detail’. Over one-fourth of respondents were not aware of open access repositories for depositing research articles.

**Table 5:** Awareness of RCUK open access policy and OA repositories

	*N*	%
*Are you aware of Research Council UK (RCUK) policy on open access?*		
Yes	721	42
No	473	28
Heard of, but no sure about the detail	522	30
Total	1716	100
*Are you aware of open*-*access repositories for depositing research articles?*		
Yes	1289	74
No	444	26
Total	1733	100

Respondents also had various beliefs of whether OA articles would receive a greater number of citations. Over half of the respondents (55%) agreed that articles that were made open-access would receive more citations, as suggested in Table 6. Another 8% disagreed with OA citation advantage and 38% chose ‘neither disagree nor agree’ indicating their uncertainty about OA citation impact.

**Table 6:** Attitudes towards citation impact of OA articles

	*N*	%
*Articles that are made open*-*access will receive more citations*		
Strongly agree	257	16
Agree	630	39
Neither disagree nor agree	621	38
Disagree	116	7
Strongly disagree	9	1
Total	1633	100

### Differences in OA publishing by university, academic discipline, gender, age and seniority

Table 7 summarises the descriptive analysis result of Gold/Green OA experience, and awareness of RCUK’s OA policy by university. As the research question is focused on experience in using OA publishing, only the selection of ‘yes’ to OA experience would be reported in Table 7, and also in Tables 8 and 9. Listing the OA experience and awareness of OA policy on the same row can also demonstrate a clear comparison. All the results (numbered as 1–4) in Table 7 are statistically significant. No 1 reports the cross tabulation between Gold OA experience and university. *p* value indicate the possibility that the observed association between Gold OA experience and university could have occurred by chance which can be called null hypothesis. The Chi square test gave an overall *p* value smaller than 0.05 (significance level at 5%), which indicates that the null hypothesis that can be rejected and there was a statistically significant association between the two variables. The significance level for No 4 was at 1% (*p* < 0.01).

**Table 7:** Experience of Gold and Green OA and awareness of RCUK OA policy by universities

University	1	2	3							4					
	Gold OA (yes)	Green OA (yes)	Gold or/and Green OA experience							Are you aware of RCUK policy on open access?					
			No		Yes to one		Both		Total	Yes		No		Heard of, but not sure about the detail	
	%	%	*N*	%	*N*	%	*N*	%	*N*	*N*	%	*N*	%	*N*	%
Cardiff University	32	31	50	52	32	33	14	15	96	44	39	33	29	35	31
Durham University	34	55	32	37	30	35	24	28	86	45	47	29	30	22	23
Imperial College London	37	43	46	41	44	39	22	20	112	46	36	38	30	43	34
King’s College London	43	33	79	47	52	31	38	22	169	68	37	67	37	47	26
Newcastle University	48	41	76	36	85	40	52	24	213	89	38	78	34	65	28
Queen’s University Belfast	31	51	17	33	26	51	8	16	51	28	54	11	21	13	25
University of Edinburgh	39	45	79	43	57	31	47	26	183	93	45	52	25	61	30
University of Glasgow	42	64	34	23	71	48	42	29	147	70	46	30	20	52	34
University of Leeds	36	43	62	40	62	40	30	19	154	75	47	33	21	50	32
University of Manchester	44	34	79	42	74	39	35	19	188	96	47	51	25	57	28
University of Warwick	33	36	21	54	9	23	9	23	39	11	26	14	33	17	40
University of York	42	47	48	36	54	41	31	23	133	53	38	31	22	55	40
Total	40	43	623	40	596	38	352	22	1571	718	42	467	27	517	30
* p* value	0.037*	0.000***	0.000***							0.007**					

Significant differences: * *p* < .05; ** *p* < .01; *** *p* < .001

**Table 8:** Experience of Gold and Green OA by academic discipline, gender, age and seniority

	Gold OA experience			Green OA experience			Gold or/and Green OA experience						
							No		Yes to one		Both		Total
	*N* (total)	*N* (yes)	%	*N* (total)	*N* (yes)	%	*N*	%	*N*	%	*N*	%	*N*
Academic discipline	**5**			**6**			**7**						
Medical and Life Sciences	579	354	61	567	231	41	168	30	223	39	176	31	567
Natural Sciences and Engineering	350	123	35	357	193	54	125	36	145	41	81	23	351
Social Sciences	426	115	27	438	180	41	204	49	147	35	69	16	420
Arts and Humanities	241	56	23	247	92	37	125	52	86	36	31	13	242
Total	1596	648	41	1609	696	43	622	39	601	38	357	23	1580
* p* value	0.000***			0.001***			0.000***						
Gender	**8**			**9**			**10**						
Female	718	276	38	727	277	38	309	44	259	37	141	20	709
Male	869	370	43	873	411	47	311	36	338	39	213	25	862
Total	1587	646	41	1600	688	43	620	39	597	38	354	23	1571
* p* value	0.001***			0.000***			0.006**						
Age	**11**			**12**			**13**						
Under 35	498	160	32	507	170	34	239	49	171	35	74	15	484
35–44	424	187	44	430	207	48	142	34	179	42	102	24	423
45–54	368	174	47	366	180	49	121	33	142	39	103	28	366
55 and over	302	126	42	302	135	45	119	39	108	36	76	25	303
Total	1592	647	41	1605	692	43	621	39	600	38	355	23	1576
* p* value	0.000***			0.000***			0.000***						
Seniority	**14**			**15**			**16**						
Researchers in training	229	59	26	238	43	18	144	66	55	25	19	9	218
Lecturers/research fellows/postdocs	634	242	38	644	290	45	238	38	268	42	127	20	633
Senior lecturers/senior researchers	268	110	41	266	123	46	102	38	103	38	63	24	268
Professors/readers	401	221	55	399	223	56	100	25	161	40	139	35	400
Total	1532	632	41	1547	679	44	584	38	587	39	348	23	1519
*p* value	0.000***			0.000***			0.000***						

Significant differences: * *p* < .05; ** *p* < .01; *** *p* < .001

**Table 9:** Experience of Gold and Green OA by awareness and attitudes towards OA publishing

	Gold OA experience			Green OA experience			Gold or/and Green OA experience						
							No		yes to one		both		Total
	*N* (total)	*N* (yes)	%	*N* (total)	*N* (yes)	%	*N*	%	*N*	%	*N*	%	*N*
*Awareness of RCUK OA policy*	**17**			**18**			**19**						
Yes	684	357	52	695	405	58	174	25	274	40	237	35	685
No	408	128	31	414	106	26	217	54	143	35	43	11	403
Heard of, but no sure about the detail	481	155	32	488	178	36	226	47	181	38	74	15	481
Total	1573	640	41	1597	689	43	617	39	598	38	354	23	1569
*p* value	0.000***			0.000***			0.000***						
*Attitudes towards citation advantage of OA articles*	**20**			**21**			**22**						
Strongly agree	237	116	49	240	106	44	79	33	92	39	64	27	235
Agree	583	246	42	591	263	45	220	38	217	37	142	25	579
Neither disagree nor agree	560	215	38	572	229	40	240	43	210	37	112	20	562
Disagree	109	33	30	112	52	46	47	42	45	40	20	18	112
Strongly disagree	9	1	11	9	4	44	5	56	3	33	1	11	9
Total	1498	611	41	1524	654	43	591	39	567	38	339	23	1497
*p* value	0.000***			0.000***			0.17						
*Attitudes towards Importance of OA publishing*	**23**			**24**			**25**						
Very important	903	414	46	909	418	46	302	34	361	40	229	26	892
Fairly important	571	205	36	585	242	41	250	44	210	37	114	20	574
Not very important	94	22	23	97	31	32	56	59	28	29	11	12	95
Not at all important	12	0	0	10	0	0	12	100	0	0	0	0	12
Total	1580	641	41	1601	691	43	620	39	599	38	354	23	1573
*p* value	0.000***			0.000***			0.000***						
*Awareness of OA Repositories*	**26**			**27**			**28**						
Yes	1195	527	44	1218	657	54	373	31	481	40	343	29	1197
No	394	118	30	392	38	10	251	65	119	31	14	4	384
Total	1589	645	41	1610	695	43	624	39	600	38	357	23	1581
*p* value	0.000***			0.000***			0.000***						

Significant differences: * *p* < .05; ** *p* < .01;*** *p* < .001

In general, respondents in universities with greater awareness of RCUK’s OA policy were also more likely to report experiences with Gold and Green OA publishing. There were some significant differences between these twelve Russell Group universities in terms of their academic staff’s experiences of OA publishing and awareness of OA policy. For example, respondents in University of Glasgow (64%) were twice more likely than respondents in Cardiff University (31%) to have deposited research papers in online repositories. Respondents in University of Glasgow were among groups with higher percentage of experience with both Gold and Green OA publishing and awareness of the RCUK’s OA policy. Academics in University of Glasgow were also among groups with lower percentage of having no experience with either Gold or Green OA publishing and answering ‘no’ to the awareness of OA policy. Respondents in Queen’s University Belfast were most likely to answer ‘yes’ (54%) to the awareness of OA policy compared to those in University of Warwick with the lowest awareness (26%). Over half of the respondents in Queen’s University Belfast (51%) had deposited research articles in online repositories compared to 36% of those in University of Warwick. Respondents in University of Warwick were also most likely to have no experience with either Gold or Green OA publishing.

Table 8 summarises the descriptive analysis results of Gold and Green OA experience by academic discipline, gender, age and job grade (seniority). All the results (numbered as 5–16) in Table 8 are statistically significant. For example, the result of No 5 indicates that the null hypothesis that discipline area and Gold OA experience are unrelated to each other can be rejected as *p* < 0.001.

The results suggest that there were significant differences between academic discipline, gender, age and seniority for using OA publishing. In general, for academics who had produced research articles, respondents in Medical and Life Sciences were most likely to publish in Gold OA journals. Those in Natural Sciences and Engineering were most likely to publish through the Green OA route. Academics in Medical and Life Sciences and Natural Sciences and Engineering were more likely to have both Gold and Green OA experience than those in Arts and Humanities and Social Sciences. Approximately half of the academics in Arts and Humanities (52%) and Social Sciences (49%) had no experience with Gold or Green OA publishing compared to 30% in Medical and Life Sciences and 36% in Natural Sciences and Engineering.

In general, men were more likely to have experience of using both Gold and Green OA publishing compared to women, although the differences were not large (less than 10%). Respondents who were older and in senior job grades had more experience of both Gold and Green OA publishing. Professors/readers were more than twice more likely to publish in OA journals and three times more likely to deposit research articles in online repositories than researchers in training. Those aged under 35 were the least likely to have published in OA journals or deposited papers in OA repositories.

### Factors associated with academics’ use of OA publishing

Table 9 summarises the descriptive analysis results of Gold and Green OA experience by awareness of OA policy and OA repositories, attitudes towards OA publishing and belief in OA citation impact. All the results (numbered as 17–28) in Table 9 except for No 22 are statistically significant. In general, academics who were aware of RCUK OA policy and OA repositories, agreeing with OA citation advantage and having positive attitudes towards OA importance were more likely to have experience of OA publishing.

Academic answered ‘yes’ to the awareness of RCUK OA policy were more likely to have both Gold and Green OA experience compared to those who answered ‘no’ or ‘heard of, but not sure about the detail’. Academics who were not aware or not sure about the detail of RCUK OA policy were more likely to have neither Gold nor Green OA experience.

Respondents who ‘strongly agreed’ or ‘agreed’ that OA articles would receive more citations were more likely than those ‘disagreed’ and ‘strongly disagreed’ to have Gold OA experience (*p* < 0.001). This pattern was not observed in Green OA experience although the *p* value was smaller than 0.001. However, when combining Gold and Green OA experience together as one variable, there was no significant association (*p* = 0.17) between that and attitudes towards OA citation advantage. Thus the null hypothesis for No 22 cannot be rejected. Respondents who selected ‘neither disagree nor agree’ were less likely to publish in OA journals compared to those who selected ‘strongly agree’ for OA citation advantage. Those who answered ‘neither disagree nor agree’ were likely to have no OA experience or have not learnt enough information about OA publishing in order to form a strong opinion.

In general, respondents who had more positive attitudes towards the importance of OA publishing were more likely to have Gold and Green OA experience and least likely to have no OA experience. Academics who were aware of OA repositories for depositing research articles were more likely to self-archive research articles and publish in OA journals.

## Discussion

This paper has shown that whilst most academics agreed with the principle of making knowledge freely available to everyone, the use of OA publishing by UK based academics was still limited despite relevant established OA policies. Barriers to OA publishing include high expenses of APCs, limited awareness of the existence of OA repositories and OA policies, uncertainty of OA articles’ citation impact and negative attitudes towards OA publishing.

Many academics would publish in OA journals only if they had no personal responsibility for paying the APCs. Similar to the findings from a study by Swan and Brown (2004) where 4% of authors of OA journal articles paid the author fee by themselves, this current study also found that 4% of respondents in our survey paid the APCs out of their own pockets for their last Gold OA papers. Many academics, especially early career researchers and PhD students, are not necessarily funded by the RCUK and thus have no access to the RCUK’s block grants to pay for APCs. Early career researchers are likely to earn less than senior researchers, and therefore, will be unable to afford APCs.

Most academics seemed to prioritise journals with a high reputation in their field rather than open access journals. The evidence suggests that the journals’ reputation and citation impact were key factors for decision making regarding submission choices, which is in line with findings from other studies (Rowlands and Nicholas 2006; Solomon and Björk 2012). The goal of OA publishing in relation to the improvement of visibility and readership has been recognised by many academics. The majority of respondents in this study agreed that open access articles would receive more citations and consequently, those academics who believed the OA journals’ citation advantage were more likely to publish in OA journals. However, since neither Gold nor Green OA publishing model has been established for very long, it is difficult to confirm whether there is a real citation advantage, this also remains a debate in the literature.

The Green OA model provides opportunities for financially disadvantaged researchers to self-archive their work if they cannot afford APCs for the Gold model. UK academics have grown less resistant towards Green OA publishing in the last eight years. As an early study with international senior academics in 2005 found only 16% of respondents had deposited an academic paper in institutional repositories and 33% indicated no intention for depositing articles, with UK authors being more ignorant of repository movement (Rowlands and Nicholas 2006). The finding of this paper suggests that institutional repositories have gained popularity and obtained more users over the years in the UK. Because 43% of respondents shared research articles in online repositories and only 8% indicated no plan to do so, it appears that UK academics are increasingly using Green OA publishing.

However, many academics still had little awareness of the existence of OA repositories and RCUK’s OA Policy. This study found that academics’ use of OA journals and OA repositories are related to their awareness of OA policy and OA repositories. Academics with less experience using OA publishing tended to be female, younger and junior. Since senior academics would have more opportunity writing funding applications to research councils, they would be more likely to know about OA repositories and RCUK’s OA policy and thus more likely to have experience using both Gold and Green OA publishing. For junior academics, if they had worked in a research team on projects that required publishing in OA journals or their senior collaborators preferred to publish through the Gold OA route, these junior members would have had Gold OA experience by default. However, early career researchers who have had no experience of applying for funding with research councils would be less likely to learn about OA publishing and related policies. There were also differences between different universities in terms of OA practice and awareness of OA policy. These differences may be related to the availability of institutional repositories for depositing research articles and how the universities promote OA policy and OA repositories to their research staff.

Women seemed to be less likely to have published in OA journal or self-archived research articles. In academia, gender inequality were evident in job status and academic achievements (Fox 2001; Hopkins et al. 2013), although some research has found no evidence to suggest discriminatory treatment of women over men for those in the same circumstances after controlling for structural, family and discipline variables (Ceci and Williams 2011). In the survey conducted for this paper, men were more likely to have senior jobs in both Sciences and Humanities discipline areas. Since men have higher job status and more research experience, they would be more likely to have experience of funding applications and thus have learned about OA policy and experienced OA publishing.

Discipline norms and culture would have influenced academics’ opportunities and preference of OA publishing. The Gold OA model seemed to be well-established amongst academics in the Medical and Life Sciences while Green OA repositories were more common in Natural Sciences and Engineering, which were in line with previous studies (Björk 2004; Björk et al. 2010). As scholars in Humanities and Social Sciences have fewer publications than colleagues in Medical and Natural Sciences, the chance of publishing through OA channels would also be lower. Since academics in the Natural Sciences are more likely to collaborate in teams than those in Social sciences and Humanities (Larivière et al. 2006), one or more of their team members may habitually deposit papers in repositories or have the funding to publish in OA journals. In terms of publication forms, academics in Humanities and Social Sciences are more likely to publish monographs and there is a lack of availability of OA journals and repositories in Humanities compared to hard science disciplines (Look and Pinter 2010). Publishing an open access monograph is very costly and requires a sustainable business model.

Attitudes towards OA publishing and OA practice are significantly associated with one another. Academics who regarded making research articles open access as important were much more likely than those who regarded OA publishing as not important to publish in OA journals and self-archive research articles. Positive attitudes might lead to more practice. On the other hand, having published in OA journal or self-archived research articles may have a positive impact on academics’ attitudes in supporting OA publishing. Academics’ use of OA journals and OA repositories are also related to their belief in OA citation advantage. Academics who felt that OA articles would receive more citations were more likely to use Gold OA publishing but not Green OA publishing. This might be because academics who published in OA journals had noticed an increase in citations and readership. However, it also suggests that those who had self-archived their research articles were not necessarily convinced that doing so would increase the citation of their articles. More studies are needed to explain the associations between belief in OA citation advantage and OA practice.

## Limitation and implications

This study drew data from a large scale survey, which was one of the first of its kind to look at UK academics’ attitudes towards open science. However the study does have limitations. The survey only sampled half of the Russell Group universities and emails were only collected from those who had a profile on their institution’s website. Those who did not have emails on the university websites would have been absent from the study because of the sampling strategy. The response rate was only 4.4%, although that is common for this type of online survey. As this study only surveyed Russell Group universities, the results cannot represent academics from all institutions in the UK. Although over half of the respondents who produced research articles reported experience of at least one of the two OA publish channels, the proportions of publications they have published in OA journal or deposited in repositories were not clear. Moreover, because it was not a random sample, the analysis cannot establish causal pathways. For example, positive personal views towards the OA citation advantage may not be the cause that someone had published in OA channels. Positive views could be caused by positive experiences of OA publishing where the authors may have noticed increased readership and citations. Future research with a random sample would provide a more representative picture of academics’ OA practice. However this will also require more manpower and funding for data collection. Robust methodologies are needed to capture academics’ various scholarly communication practices over time.

As the research assessments after the 2014 REF requires research outputs accepted for publication after 1 April 2016 to comply with HEFCE’s open access policy, it is clear that open access publishing is going to be a major factor in UK academia. The awareness of OA policy and OA repositories is highly important for enhancing the use of Gold and Green OA. The survey results suggested that many academics had little awareness of the existence of OA repositories or RCUK’s OA policy. There were also significant differences between academics from various universities in terms of their awareness of OA policy and experience with OA publishing. Respondents in universities with greater awareness of RCUK’s OA policy were more likely to report experiences with Gold and Green OA publishing. This indicates that the promotion for OA policy for some Russell Groups institutions had not yet been enforced. Institutions with their own OA repositories need to promote their service to be known and understood by more academics if they want their service to be fully used. Institutions without OA repositories need to find alternative methods to help their academic staff comply with RCUK and HEFCE’s OA policies in the future. As HEFCE’s OA policy is related to the research assessments post 2014 REF, UK higher education institutions need to promote OA policy in order to ensure that it is known and understood by academics including those who are not funded by RCUK or HEFCE but who might be in the future. Institutions should reinforce these OA polices to academics through various channels including training especially for those at the start of their careers such as PhD students and research assistants who are more likely to be unaware of those policies as suggested by the findings of this study.

This study found that academics who had self-archived their research articles were not necessarily convinced that doing so would increase the number of citations. Incentives seem to be very important to academics’ publishing practice. Citation impact is highly relevant to academics’ career advancement and it is clear more robust analysis is needed for investigating the citation effects of OA articles. Other impacts of OA publishing need to be studied. For example, has the increase of OA availability helped the public learn about science and improved the public’s satisfaction with science communication? Once positive and reliable impacts of OA publishing are found, funders and academic institutions need to inform academics along with mandatory OA policies.

One of the concerns of publishing in Gold OA is the quality of new OA journals. Since the quality of new OA journals may still be uncertain to many academics, research funding bodies could have guidelines for what kind of journals academics should or should not consider. It is clear that open access journals need to be quality assured in the same way that closed access journals are, including the peer review process.

This paper provides some insights into the question of who have supported OA publishing. A further question for follow-up research may be how to help researchers better understand and use OA publishing.

## Footnotes

1
http://www.bristol.ac.uk/red/research-policy/pure/. Accessed 23 January 2016.

2
http://www.bodleian.ox.ac.uk/ora/deposit-in-ora. Accessed 23 January 2016.

3 arXiv.org started in 14 August 1991 as hep-th@xxx.lanl.gov. See http://arxiv.org/abs/1108.2700 (accessed 18 May 2015).

## References

[CR1] Barnes S, Lewin C, Somekh B, Lewin C (2005). An introduction to inferential statistics: Testing for differences and relationships. Research methods in the social sciences.

[CR2] Björk BC (2004). Open access to scientific publications—An analysis of the barriers to change?. Information Research.

[CR3] Björk BC, Welling P, Laakso M, Majlender P, Hedlund T, Guonason G (2010). Open Access to the scientific journal literature: Situation 2009. PLoS ONE.

[CR4] Bohannon J (2013). Who’s afraid of peer review?. Science.

[CR5] Børing, P., Flanagan, K., Kaloudis, A., & Karakasidou, A. (2010). Mobility survey of the higher education sector. Report. Retrieved 5 January, 2015, from https://www.escholar.manchester.ac.uk/uk-ac-man-scw:158492/

[CR6] Bucchi M (2004). Science in society: An introduction to social studies of science.

[CR7] Calver MC, Bradley JS (2010). Patterns of citations of open access and non-open access conservation biology journal papers and book chapters. Conservation Biology.

[CR8] Ceci SJ, Williams WM (2011). Understanding current causes of women’s underrepresentation in science. Proceedings of the National Academy of Sciences.

[CR9] Correia AMR, Teixeira JC (2005). Reforming scholarly publishing and knowledge communication: From the advent of the scholarly journal to the challenges of open access. Online Information Review.

[CR10] Craig ID, Plume AM, McVeigh ME, Pringle J, Amin M (2007). Do open access articles have greater citation impact? A critical review of the literature. Journal of Informetrics.

[CR11] Crow, J. (2008). Open access and scholarly communication. Retrieved February 24, 2017 from http://eprints.rclis.org/12510/

[CR12] Darley, R., Reynolds, D., & Wickham, C. (2014). Open access journals in humanities and social science: A British Academy research project. Retrieved 24/04/2014, from http://www.britac.ac.uk/openaccess/index.cfm

[CR13] Davies SR (2008). Constructing communication: Talking to scientists about talking to the public. Science Communication.

[CR14] Davis PM (2009). Author-choice open-access publishing in the biological and medical literature: A citation analysis. Journal of the American Society for Information Science and Technology.

[CR15] Davis PM (2010). Does open access lead to increased readership and citation. The Physiologist.

[CR16] Davis PM (2011). Open access, readership, citations: A randomized controlled trial of scientific journal publishing. The FASEB Journal.

[CR17] Davis PM, Fromerth MJ (2007). Does the arXiv lead to higher citations and reduced publisher downloads for mathematics articles?. Scientometrics.

[CR18] Davis PM, Lewenstein BV, Simon DH, Booth JG, Connolly MJL (2008). Open access publishing, article downloads, and citations: Randomised controlled trial. British Medical Journal.

[CR19] Davis PM, Walters WH (2011). The impact of free access to the scientific literature: A review of recent research. Journal of the Medical Library Association.

[CR20] Dutton, W., Di Gennaro, C., & Millwood Hargrave, A. (2005). Oxford Internet Survey 2005 report: The internet in Britain. *Available at SSRN 1327035*.

[CR21] Eysenbach G (2006). Citation advantage of open access articles. PLoS Biology.

[CR22] Field A (2009). Discovering statistics using SPSS.

[CR23] Fox MF (2001). Women, science, and academia graduate education and careers. Gender & Society.

[CR24] Gaule P, Maystre N (2011). Getting cited: Does open access help?. Research Policy.

[CR25] Gray J, Hey T, Tansley S, Tolle K (2009). Jim Gray on eScience: A transformed scientific method. The fourth paradigm: Data-intensive scientific discovery.

[CR26] Hardigan PC, Succar CT, Fleisher JM (2012). An analysis of response rate and economic costs between mail and web-based surveys among practicing dentists: A randomized trial. Journal of Community Health.

[CR27] Harnad S, Brody T, Vallières F, Carr L, Hitchcock S, Gingras Y (2004). The access/impact problem and the green and gold roads to open access. Serials Review.

[CR28] HEFCE. (2015). Policy for open access in the post-2014 research excellence framework: Updated July 2015. Retrieved 31 October 2015, from http://www.hefce.ac.uk/pubs/year/2014/201407/

[CR29] Helsper EJ, Eynon R (2010). Digital natives: Where is the evidence?. British educational research journal.

[CR30] Henneken, E. A., Kurtz, M. J., Eichhorn, G., Accomazzi, A., Grant, C., Thompson, D., & Murray, S. S. (2006). Effect of e-printing on citation rates in astronomy and physics. *Journal of Electronic Publishing, 9*(2). doi:10.3998/3336451.0009.202.

[CR31] HESA. (2013). Higher education statistics for the United Kingdom 2011/12 Retrieved 21 September, 2013, from http://www.hesa.ac.uk/

[CR32] Hopkins AL, Jawitz JW, McCarty C, Goldman A, Basu NB (2013). Disparities in publication patterns by gender, race and ethnicity based on a survey of a random sample of authors. Scientometrics.

[CR33] Kim J (2011). Motivations of faculty self-archiving in institutional repositories. Journal of Academic Librarianship.

[CR34] Kurtz MJ, Eichhorn G, Accomazzi A, Grant C, Demleitner M, Henneken E, Murray SS (2005). The effect of use and access on citations. Information Processing and Management.

[CR35] Laakso M (2014). Green open access policies of scholarly journal publishers: A study of what, when, and where self-archiving is allowed. Scientometrics.

[CR36] Laakso M, Welling P, Bukvova H, Nyman L, Björk BC, Hedlund T (2011). The development of open access journal publishing from 1993 to 2009. PLoS ONE.

[CR37] Larivière V, Gingras Y, Archambault É (2006). Canadian collaboration networks: A comparative analysis of the natural sciences, social sciences and the humanities. Scientometrics.

[CR38] Lawrence S (2001). Free online availability substantially increases a paper’s impact. Nature.

[CR39] Lewin C, Somekh B, Lewin C (2005). Elementary quantitative methods. Research methods in the social sciences.

[CR40] Look H, Pinter F (2010). Open access and humanities and social science monograph publishing. New Review of Academic Librarianship.

[CR41] McCabe M, Snyder CM (2014). Identifying the effect of open access on citations using a panel of science journals. Economic Inquiry.

[CR42] McGuigan, G., & Russel, R. (2008). The business of academic publishing: A strategic analysis of the academic journal publishing industry and its impact on the future of scholarly publishing. *The Electronic Journal of Academic and Special Librarianship, 9*(3). Retrieved February 24, 2017 from http://southernlibrarianship.icaap.org/content/v09n03/mcguigan_g01.html

[CR43] Metcalfe TS (2005). The rise and citation impact of astro-ph in major journals. Bulletin of the American Astronomical Society.

[CR44] Metcalfe TS (2006). The citation impact of digital preprint archives for solar physics papers. Solar Physics.

[CR45] Moed HF (2007). The effect of “open access” on citation impact: An analysis of ArXiv’s condensed matter section. Journal of the American Society for Information Science and Technology.

[CR46] Moser CA, Kalton G (2004). Survey methods in social investigation.

[CR47] Nicholas D, Clark D, Rowlands I, Jamali HR (2009). Online use and information seeking behaviour: Institutional and subject comparisons of UK researchers. Journal of Information Science.

[CR48] Nicholas D, Rowlands I (2011). Social media use in the research workflow. Information Services and Use.

[CR49] Pham N, Bogdan M (2010). Automatic data mining on internet by using PERL. Industrial Communication Systems.

[CR50] Pinfield, S. (2003). Open archives and UK institutions: An overview. *D*-*lib Magazine*. Retrieved from: http://www.dlib.org/dlib/march03/pinfield/03pinfield.html

[CR51] Procter R, Williams R, Stewart J, Poschen M, Snee H, Voss A, Asgari-Targhi M (2010). Adoption and use of Web 20 in scholarly communications. Philosophical Transactions of the Royal Society A.

[CR52] RCUK. (2013). RCUK policy on open access. Retrieved 21 November 2014, from http://www.rcuk.ac.uk/research/openaccess/

[CR53] Rowlands I, Nicholas D (2006). The changing scholarly communication landscape: An international survey of senior researchers. Learned Publishing.

[CR54] RussellGroup. (2016). Facts and figures. Retrieved 15 January 2016, from http://russellgroup.ac.uk/about/facts-and-figures/

[CR55] Schwarz, G. J., & Kennicutt, R. C. J. (2004). Demographic and citation trends in astrophysical journal papers and preprints. *Arxiv preprint* arXiv:astro-ph/0411275.

[CR56] Solomon DJ, Björk BC (2012). Publication fees in open access publishing: Sources of funding and factors influencing choice of journal. Journal of the American Society for Information Science and Technology.

[CR57] Sotudeh H, Ghasempour Z, Yaghtin M (2015). The citation advantage of author-pays model: The case of Springer and Elsevier OA journals. Scientometrics.

[CR58] Swan A, Jacobs N (2006). The culture of open access: Researchers’ views and responses. Open access: Key strategic.

[CR59] Swan A, Brown S (2004). Authors and open access publishing. Learned Publishing.

[CR60] Thompson JW (2002). The death of the scholarly monograph in the Humanities? Citation Patterns in Literary Scholarship. Libri.

[CR61] Wang X, Liu C, Mao W, Fang Z (2015). The open access advantage considering citation, article usage and social media attention. Scientometrics.

[CR62] Weisberg HF, Bowen BD (1977). An introduction to survey research and data analysis.

[CR63] Williams P, Stevenson I, Nicholas D, Watkinson A, Rowlands I (2009). The role and future of the monograph in arts and humanities research. Aslib Proceedings.

[CR64] Willinsky J (2010). The stratified economics of open access. OJS på dansk.

[CR65] Xia JF (2007). Assessment of self-archiving in institutional repositories: Across disciplines. Journal of Academic Librarianship.

[CR66] Xia JF, Sun L (2007). Assessment of self-archiving in institutional repositories: Depositorship and full-text availability. Serials Review.

